# Imperforate Hymen and Hematometrocolpos in a Female With Back Pain and Urinary Retention

**DOI:** 10.7759/cureus.30525

**Published:** 2022-10-20

**Authors:** Ojeagbase Asikhia, Muhammad Durrani, Carla Dugas, Curt Cackovic, Brian Jerusik

**Affiliations:** 1 Department of Emergency Medicine, Inspira Medical Center, Vineland, USA

**Keywords:** back pain, pediatric, ultrasound, hematometrocolpos, imperforate hymen

## Abstract

A 12-year-old female with primary amenorrhea presented to the emergency department with episodic low back pain, suprapubic discomfort, and acute urinary retention. A protruding purple mass was noted at the vaginal introitus consistent with an imperforate hymen. Point-of-care bedside transabdominal ultrasonography revealed a distended uterus containing hypoechoic material. The patient underwent formal pelvic ultrasonography, which revealed a markedly enlarged uterus containing a large number of blood products, thinned myometrium, and a distended vaginal canal consistent with hematometrocolpos secondary to imperforate hymen. Imperforate hymen is a rare congenital anomaly of the female urogenital tract, in which the hymen obstructs the vaginal opening causing a vaginal outlet obstruction. Vaginal outlet obstruction secondary to imperforate hymen may lead to retrograde menstruation with a collection of blood within the uterus and vagina, which is termed hematometrocolpos. Treatment is based on identifying and treating the underlying imperforate hymen with surgical approaches. The growing use of bedside ultrasonography allows the clinician to rapidly and accurately diagnose hematometrocolpos. The use of point-of-care bedside ultrasonography can serve as an essential tool as delayed diagnosis and treatment of this rare condition are associated with significant morbidity and lifelong infertility.

## Introduction

Imperforate hymen is a rare congenital anomaly of the female urogenital tract, in which the hymen obstructs the vaginal opening causing a complete or partial vaginal outlet obstruction. It is thought to result from the failure of appropriate urogenital sinus development where the hymenal membrane does not undergo degeneration and persists around the vaginal introitus [1]. It may be readily visualized in neonates as a bulging hymen with a blue hue on examination of the external genital area due to a collection of mucoid material in the vaginal canal from endogenous maternal estrogen stimulation. This mucoid collection is commonly reabsorbed in the neonatal period, and the diagnosis is subsequently overlooked with patients having an asymptomatic state until the onset of menarche. After menarche, patients may experience vaginal outlet obstruction secondary to imperforate hymen causing retrograde menstruation of blood within the uterus (hematometria) and vagina (hematocolpos) or both (hematometrocolpos).

The main clinical presentation of imperforate hymen is typically primary amenorrhea as well as cyclic abdominal pain and abdominal distension [2]. Without proper identification and treatment, symptoms may progress to obstructive uropathy, acute renal failure, and infected retained vaginal blood products. Treatment is based on identifying and treating the underlying imperforate hymen with surgical approaches and subsequent drainage of the hematometrocolpos. Delayed treatment or missed diagnosis is associated with chronic pain, endometriosis, adhesions, and lifelong infertility [3]. This case report serves to add to the body of literature on imperforate hymen and to further reinforce the role of bedside ultrasonography in identifying secondary signs of imperforate hymen.

## Case presentation

**Figure 1:**
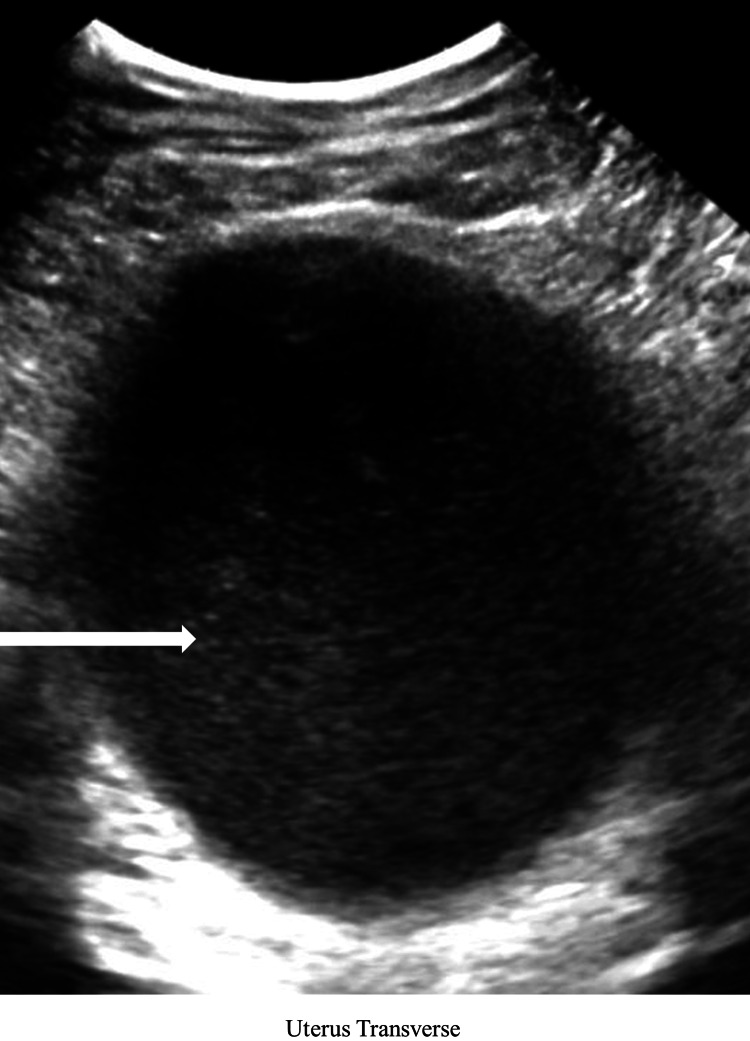
Transverse orientation of the distended uterus containing a large amount of hypoechoic blood products (white arrow)

**Figure 2:**
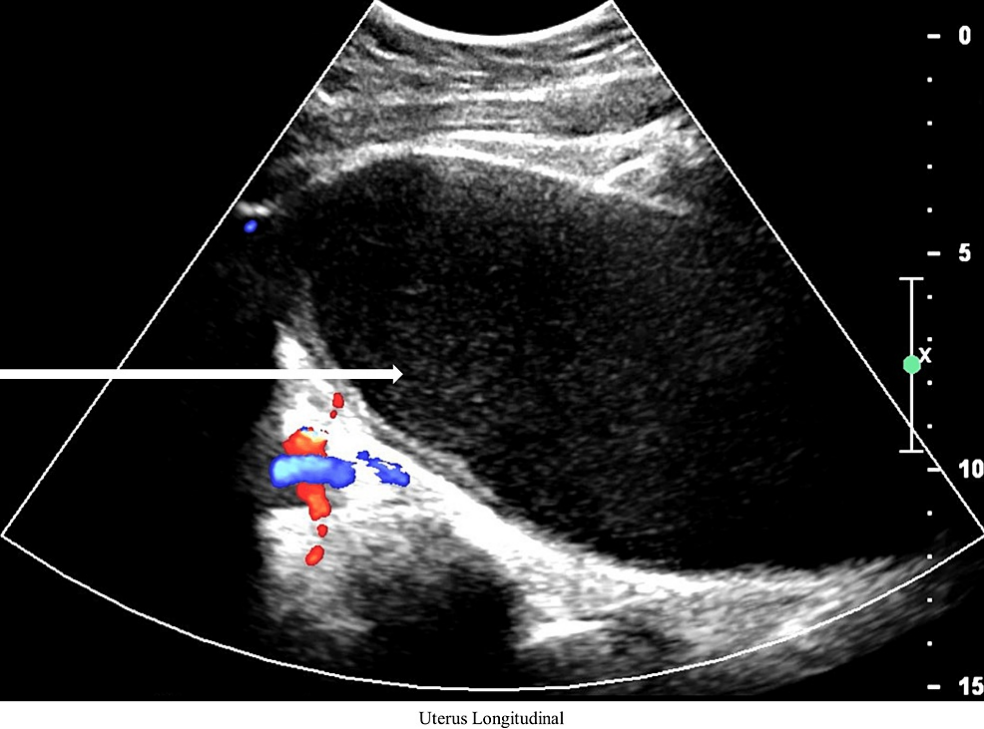
Longitudinal orientation of the distended uterus containing a large amount of hypoechoic blood products (white arrow)

A 12-year-old female presented with episodic lower back pain over the previous three months along with the two-day onset of suprapubic discomfort. The patient had no previous medical history, but it was noted that she had not reached menarche despite secondary sex characteristic development. The patient presented with normal vital signs, and examination revealed mild suprapubic distension along with the presence of a purple mass at the vaginal introitus consistent with imperforate hymen. A bladder scan revealed acute urinary retention with 650 ml of urine. Laboratory workup included a complete blood count, complete metabolic panel, urinalysis, and pregnancy testing, which were found to be unexceptional with a negative pregnancy test. Bedside transabdominal ultrasound revealed a distended uterus with internal hypoechoic material prompting formal pelvic ultrasonography. Pelvic ultrasonography revealed a distended vaginal canal, a markedly enlarged uterus containing a large number of blood products, and thinned myometrium consistent with hematometrocolpos secondary to imperforate hymen (Figures 1, 2).

## Discussion

Pelvic ultrasonography confirmed a diagnosis of hematometrocolpos secondary to imperforate hymen. The patient subsequently required placement of a foley catheter and underwent a successful X-shaped hymenotomy allowing for drainage of the hematometrocolpos. Imperforate hymen is an uncommon urogenital tract fusion anomaly causing complete obstruction of the vaginal orifice with an incidence rate of 0.05%-0.1% [1]. The obstruction causes accumulation of uterine and vaginal secretions as well as retrograde menstruation [1]. The clinical manifestations are variable and include back pain, cyclic abdominal pain, urinary retention, abdominal masses, protruding hymen, primary amenorrhea, urinary tract infection, and renal failure [2]. Patients often present with primary amenorrhea two to three years after initial breast development.

Cases are typically diagnosed after menarche due to symptoms manifesting as a result of the retrograde accumulation of blood in the vagina and uterus during menstruation [3,4]. The imperforate hymen may manifest with retained blood within the uterus (hematometria), vagina (hematocolpos), or both (hematometrocolpos) [5]. Diagnosis of hematometrocolpos can be made with ultrasonography revealing hypoechoic material within a distended uterus and vaginal canal. The use of point-of-care bedside ultrasonography presents a unique opportunity for the clinician to diagnose hematometrocolpos as a secondary complication of the imperforate hymen in the right clinical setting. This is especially helpful as the initial symptoms may be present in a wide range of pathologies, and the proper utilization of bedside ultrasonography may help to rapidly and accurately narrow the differential diagnosis. Treatment is based on identifying and treating the underlying imperforate hymen with surgical approaches and subsequent drainage of the hematometrocolpos [5].

## Conclusions

We concluded that this patient’s episodic low back pain and urinary retention were a result of retained blood products in the uterus and vagina secondary to an undiagnosed imperforate hymen. We suspected the diagnosis by the fact that the patient had developed secondary sex characteristics despite not reaching menarche. This allowed for a targeted physical examination that revealed the characteristic vaginal mass consistent with an imperforate hymen. Furthermore, the utilization of bedside ultrasonography allowed for rapid identification of blood products and aided in confirming the diagnosis while avoiding the use of radiation associated with computerized tomography scanning. Imperforate hymen can have a varied presentation and is associated with significant morbidity and the potential for lifelong infertility. This necessitates increased awareness and vigilance on part of the clinician. This case report should serve to reinforce the clinical presentation and diagnosis of imperforate hymen. We hope this case adds to the body of literature on imperforate hymen as well as reinforces the utility of point-of-care ultrasonography in diagnosing this condition.
